# DNA damage induction and repair in peripheral blood mononuclear cells induced by internal *ex vivo* irradiation with β- and γ-emitting radionuclides^☆^

**DOI:** 10.1016/j.zemedi.2026.03.010

**Published:** 2026-03-22

**Authors:** Sarah Schumann, Isabella Strobel, Johanna Hahn, Alberto Meca Zapata, Michael Lassmann, Harry Scherthan, Uta Eberlein

**Affiliations:** a Department of Nuclear Medicine, University of Würzburg, Würzburg, Germany; b Bundeswehr Institute of Radiobiology affiliated to the University of Ulm, Munich, Germany

**Keywords:** 53BP1, Biodosimetry, DSB repair, γ-H2AX, Internal irradiation, Nuclear medicine

## Abstract

Purpose: Aim of the study was to investigate DNA damage induction and repair in peripheral blood mononuclear cells (PBMCs) after internal *ex vivo* irradiation with short-lived radionuclides with varying emission properties.

Methods: Blood samples from healthy volunteers were irradiated with different activity concentrations for 1 h, resulting in absorbed doses to the blood from nominally 3 to 100 mGy. DNA double-strand breaks (DSBs) in PBMCs were assessed by quantifying radiation-induced γ-H2AX+53BP1-positive foci (RIF). In part A of the study, four different radionuclides (^177^Lu, ^90^Y, ^99m^Tc and ^68^Ga) were used to test for radionuclide dependence. In part B, blood samples were exposed to ^177^Lu and cells were fixed at three different time points (directly, 4 h and 24 h after irradiation) to investigate DSB repair and its dependence on the absorbed dose.

Results: The number of RIF increases linearly with the absorbed dose to the blood, independent of the radionuclide used for irradiation. The decline in RIF after irradiation can be described by an exponential function, with a trend towards higher repair rates at higher absorbed doses to the blood, i.e. (0.20±0.12) h⁻^1^ for 25 mGy, (0.22±0.04) h⁻^1^ for 50 mGy, and (0.37±0.06) h⁻^1^ for 100 mGy.

Conclusion: Our results show a clear relationship between absorbed dose and DSB foci induced by internal irradiation in blood cells, independent of the emission properties of the particular radionuclide used. A better understanding of DNA damage repair dynamics after internal irradiation can improve future nuclear medicine therapies.

## Introduction

Nuclear medicine diagnostics or radiopharmaceutical therapies (RPTs) deploy a wide range of radionuclides with different emission properties. The hematopoietic system is, for many therapeutic applications, one of the organs-at-risk. As the absorbed dose to the blood is a potential surrogate for the absorbed dose to the bone marrow, in-depth knowledge of the biokinetics of the radiopharmaceutical and the exposure of blood cells is important [1], [2].

Currently, most RPTs are based on administering a fixed activity and do not involve individual dosimetry [3]. Although absorbed dose-effect relationships for these therapies have not been fully exploited to date, they are expected to help personalize and improve nuclear medicine therapies.

Ionizing radiation is known to damage nuclear DNA of which the DNA double-strand break (DSB) is considered to be a severe lesion threatening cellular survival and genome integrity. DSBs are associated with microscopically visible foci of γ-H2AX and 53BP1 DSB-repair-associated proteins and represent an interesting biological endpoint for biodosimetry [4]. They can be reliably quantified using the γ-H2AX+53BP1 DSB focus assay, particularly in blood cells, and especially in the low-dose range below 100 mGy [5], [6]. The number of radiation-induced γ-H2AX+53BP1 foci (RIF) that can be counted in the cells represents the number of DSBs. It has already been shown in several studies, both *ex vivo* and *in vivo*, that it is feasible to use the assay to describe the induction and decline of DSBs in peripheral blood mononuclear cells (PBMCs) during and after internal irradiation with radionuclides [6], [7], [8], [9], [10], [11], [12], [13], [14], [15], [16], [17].

The advantage of an *ex vivo* setup is that experiments can be conducted under well-controlled conditions. For example, this makes it possible to irradiate blood samples with different radionuclides to achieve predefined absorbed dose values, enabling direct comparisons.

Therefore, we have chosen an *ex vivo* setup for this study to investigate the following unanswered questions:

1. Are there radionuclide-dependent differences in the induction of RIF in PBMCs?

2. Does the decrease in RIF in PBMCs after internal irradiation depend on the absorbed dose to the blood?

## Materials and methods

### Subjects and experimental setup

The study consists of two parts. Part A focuses on investigating the influence of different radionuclides on the induction of DSBs. For this purpose, 16 experimental series with four healthy volunteers V1 – V4 (2 male and 2 female, aged between 28 and 61 years) and four different radionuclides were performed. Part B was designed to investigate the decline in DSBs following internal irradiation with ^177^Lu and thus the repair kinetics. For this, eight experimental series with eight healthy volunteers V2, V3 and V4 (also included in part A) and V5, V6, V7, V8 and V9 (4 male and 4 female, aged between 23 and 40 years) were performed.

For each test series, blood was taken from each volunteer using Li-Heparin blood collecting tubes (S-Monovette®, Sarstedt AG & co. KG, Nümbrecht, Germany) and divided into five to seven aliquots, which were then internally irradiated by adding radionuclide solutions with different activity concentrations, resulting in different absorbed doses to the blood from nominally 3 mGy up to 100 mGy.

### Internal irradiation and preparation of blood samples

Four β- and γ-emitting radionuclides with different emission properties, which are used in RPTs and nuclear medicine diagnostics, were selected for the *ex vivo* irradiation: the β^-^-emitters ^177^Lu ([^177^Lu]Lu-DOTATATE or [^177^Lu]LuCl_3_, depending on availability) and ^90^Y ([^90^Y]YCl_3_), the β^+^-emitter ^68^Ga ([^68^Ga]GaCl_3_) and the γ-emitter ^99m^Tc ([^99m^Tc]NaTcO_4_). By adding either phosphate buffered saline (PBS) or sodium chloride (NaCl) to the radiopharmaceuticals, stock solutions with pre-defined activity concentrations were prepared for the internal irradiation of the blood samples. 1 ml of radioactive solution was added for each irradiation, containing nominally 0.32 – 5.40 MBq ^177^Lu (for protocol A) or 0.30 – 10.00 MBq ^177^Lu (for protocol B) or 0.07 – 0.92 MBq ^90^Y (for protocol A) or 0.11 – 1.81 MBq ^68^Ga (for protocol A) or 2.65 – 44.12 MBq ^99m^Tc (for protocol A) in order to achieve the pre-defined absorbed dose values after one hour irradiation.

Protocol A: Aliquots of 3.5 ml blood were pipetted in round-bottom tubes (No. 55.526.006, Sarstedt AG & Co. KG, Nümbrecht, Germany), supplemented with 1 ml radioactive solution and incubated at 37°C on a roller-mixer to ensure uniform irradiation. The irradiation duration was 1 h. Directly afterwards, the blood was transferred to CPT tubes (BD Vacutainer® CPT™ Mononuclear Cell Preparation Tube, BD, Heidelberg, Germany) and centrifuged according to the manufacturer’s instructions in order to separate PBMCs from the radioactive solution and the rest of the blood. The cells were washed twice in PBS before they were fixed in a 70% ethanol solution. For background foci detection, one aliquot per experimental series was prepared without adding activity following the same protocol, i.e. as non-irradiated baseline sample.

Protocol B corresponds to protocol A with the following changes: The aliquots contained 7 ml blood supplemented with 1 ml ^177^Lu solution and were pipetted into an 8 ml vial with a screw cap (No. 60.542.007, Sarstedt AG & Co. KG, Nümbrecht, Germany). After the separation, PBMCs were split in three parts: One part of the cells was processed and directly (d) fixed in ethanol as in part A, while RPMI was added to the other two parts after the second wash followed by 4 h and 24 h cell culture at 37°C in an incubator, before they were fixed in 70% ethanol.

### Immunofluorescent staining and evaluation of DNA damage

After ethanol-fixation, PBMCs were immunofluorescently stained with γ-H2AX and 53BP1 antibodies as described by Scherthan *et al.* [18]. An experienced investigator (HS) counted co-localizing γ-H2AX and 53BP1 foci in 100 PBMC nuclei per sample in all experiments, to ensure comparability. Corresponding counting errors were calculated assuming a Poisson distribution. To determine the number of radiation-induced foci (RIF), the number of foci in the corresponding non-irradiated sample of the same experimental series, i.e. the individual baseline foci value, was subtracted and an error propagation was performed.

### Blood dosimetry

Absorbed doses to the blood $D_{B\mathrm{lood}}\left(T_{I}\right)$ were calculated according to the MIRD formalism, assuming that blood is both the source and target organ:

(1) $$\begin{array}{c} D_{B\mathrm{lood}}\left(T_{I}\right)=A^{*}\cdot d_{\mathrm{Blood}}\left(T_{I}\right)\\ \text{with}\;d_{\mathrm{Blood}}\left(T_{I}\right)=\lambda^{-1}\cdot \left(1-\exp\left(-\lambda \cdot T_{I}\right)\right)\cdot S_{\mathrm{Blood}} \end{array}$$

The incubation time $T_{I}$ in this study is 1 hour, and the decay constant λ refers exclusively to the physical half-life of the respective radionuclide, i.e. $\lambda =\lambda_{\mathrm{phys}}$.

The S-values $S_{B\mathrm{lood}}$ for ^68^Ga, ^99m^Tc and ^90^Y were calculated by adapting data on energy deposition in blood vessels of different sizes published by Hänscheid *et al.* [19] to the geometry of the incubation tube used in Part A (with an inner diameter of 4.75 mm). For ^177^Lu, S-values $S_{\mathrm{Blood}}$ were determined by Monte Carlo simulations for the exact geometry of the incubation tubes and have been published by Eberlein *et al.* [6] for the round-bottom tube used in Part A and by Salas-Ramirez *et al.* [20] for the vial used in Part B. Based on the S-values, absorbed dose coefficients $d_{\mathrm{Blood}}\left(T_{I}\right)$ for $T_{I}=1h$ were calculated (Supplementary Table 1).

To determine the activity concentration $A^{*}$, an aliquot of each irradiated blood sample was measured in a calibrated high purity germanium detector (Canberra, Rüsselsheim, Germany). γ-emission lines were evaluated for the samples irradiated with ^177^Lu (at 208.4 keV), with ^68^Ga (at 511 keV) and with ^99m^Tc (at 140.5 keV). For the samples irradiated with ^90^Y, the γ-emission line at 511 keV resulting from annihilation of electron-positron pairs was evaluated. However, due to the low emission probability (6.38E-5 per decay), a reliable quantification of the activities < 200 kBq in the individual blood aliquots was not possible. Instead, part of the ^90^Y stock solution prepared for irradiation of the blood samples was measured before it was added to the blood and its activity concentration was determined. The activity concentration in the blood was then calculated based on the added volume. For two experimental series of part B, the germanium detector was not available, so measurements were carried out in a calibrated well counter (Canberra, Rüsselsheim, Germany).

In order to account for uncertainties in the calculation of the absorbed dose to the blood, error propagation was performed. The activity, the time between the addition of the activity and the measurement in the germanium detector, the volume of the measured sample, and the absorbed dose coefficient were taken into account as independent variables, with respective uncertainties. The uncertainty of the activity was estimated assuming a Poisson distribution. For the uncertainties of the time difference and the volume, 1 min and 0.5 µl were assumed, respectively. The uncertainty of the absorbed dose coefficient was estimated at 1% to 2%, depending on the radionuclide used. Since no individual measurements were available for determining the activity in the samples irradiated with ^90^Y, a fixed uncertainty of 10% was assumed for the experimental series with ^90^Y.

### Modelling of DNA damage repair

An approach to describe the time-dependent decline of RIF is described in an earlier publication [14]. Accordingly, the average number of RIF N(t) at time t is described by a monoexponential decay function:

(2) $$N\left(t\right)=N_{0}\cdot \left(\left(1-Q\right)\cdot \exp\left(-R\cdot t\right)+Q\right)$$

Here, $N_{0}$ is the maximum number of RIF per cell, Q is the fraction of unrepaired RIF (i.e. the residual damage) and R denotes the repair rate in h^-1^.

### Statistical analysis

OriginPro (OriginLab Corporation) was used for data visualization, fitting and statistical analysis. To test whether two normally distributed data sets were significantly different, t-tests were performed. If normal distribution could not be confirmed, Mann-Whitney U tests or Wilcoxon signed-rank tests were applied. The significance level was generally set at 5%.

## Results

### S-values and absorbed doses to the blood

The S-values and absorbed dose coefficients that were calculated for the four radionuclides ^177^Lu, ^90^Y, ^68^Ga, and ^99m^Tc are listed in Supplementary Table 1. The absorbed doses to the blood ranged from 5.5 mGy to 109.2 mGy in all irradiated samples of part A and from 2.4 mGy to 101.9 mGy in all irradiated samples of part B.

### Results of part A: Absorbed dose-dependent induction of RIF for different radionuclides

**Fig. 1:**
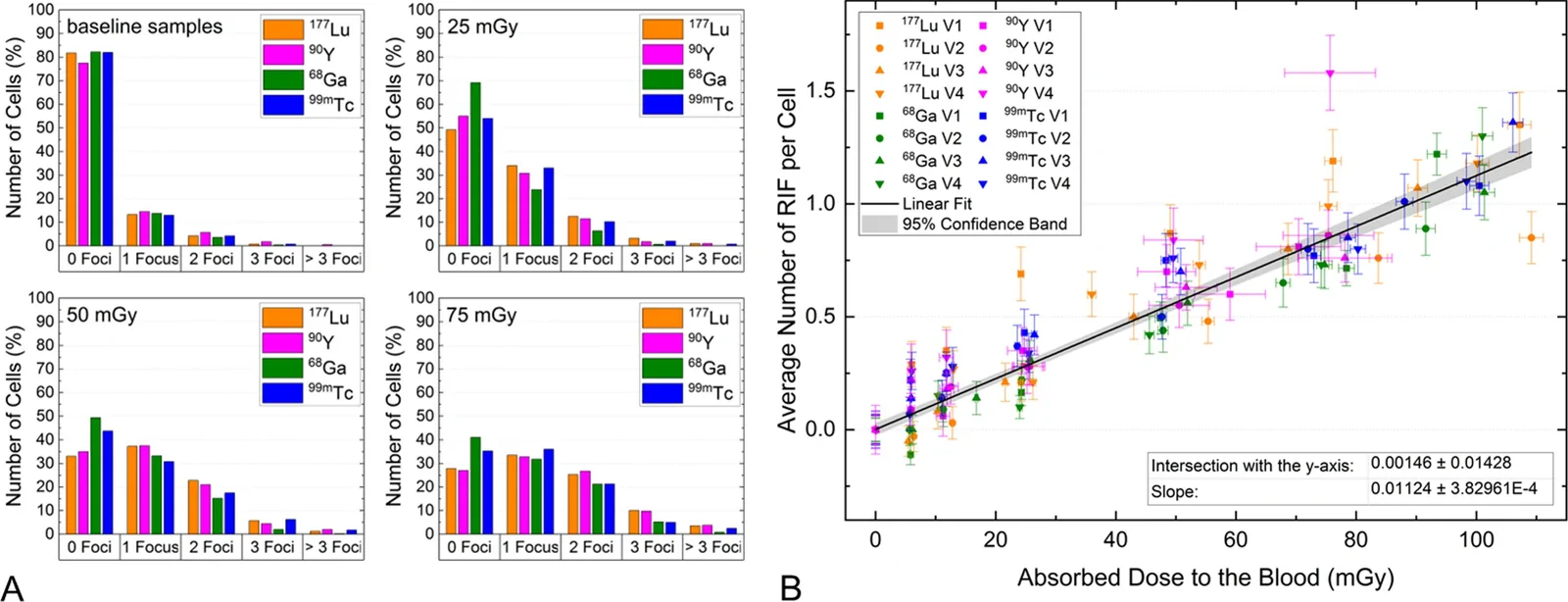
A: Percentage of cells with n γ-H2AX+53BP1 DSB foci depending on the absorbed dose to the blood (0 mGy, 25 mGy, 50 mGy and 75 mGy) and the radionuclide used in the various samples. For the non-irradiated baseline samples (0 mGy), the radionuclide is specified solely for assignment to the corresponding experimental series. B: Average number of RIF per cell as a function of the absorbed dose to the blood with a linear fit to the pooled data (black line) and 95% confidence band (grey area). Adapted from [30].

In the non-irradiated baseline samples, 81% of the cell nuclei did not show any foci. Two or more foci per cell were detected only in less than 6% of the cells. After irradiation with nominally $D_{\mathrm{Bl}ood}=50\;mGy$, 40% of the cell nuclei did not show any foci. Two or more foci per cell were detected in 25% of the cells (Fig. 1A).

Fig. 1B shows the average number of RIF per cell as a function of the absorbed dose to the blood. Consistent with the published data of Eberlein *et al.* [6], an increase in the average number of RIF with increasing absorbed dose to the blood was observed, and this increase is best represented by a linear function.

To investigate whether there were nuclide- or subject-specific differences in the induction of RIF, individual linear fits were performed for each of the 16 experimental series, and the slopes were tested for significant differences. The resulting fit parameters of the individual fits are shown in Supplementary Table 2. To test whether the induction of RIF was subject-dependent, the slopes were sorted by volunteer and normality was confirmed. Independent t-tests revealed no significant difference in the means of the four groups. Similarly, the slopes were sorted by the radionuclides to check for a nuclide dependence of the induction of the RIF. Since a normal distribution could not be confirmed for one of the four groups, the Mann-Whitney U test was used for comparison in this case, revealing no significant difference between the groups. Thus, the induction of RIF in our study was neither dependent on a particular volunteer nor on the radionuclide used for irradiation.

Therefore, all data points were pooled and one linear fit was performed to establish a general *ex vivo* calibration curve describing the average number of RIF per cell N as a function of the absorbed dose to the blood $D_{\mathrm{Bl}ood}$. The resulting linear regression line including a 95% confidence band is shown in Fig. 1B. The linear equation of the calibration curve (r = 0.94) is given by:

(3) $$N\left(D_{\mathrm{Bl}ood}\right)=\left(0.0112\pm 0.0004\right)\;mGy^{-1}\cdot D_{\mathrm{Bl}ood}+\left(0.0015\pm 0.0154\right)$$

### Results of part B: Absorbed dose-dependent decrease of RIF over time

**Fig. 2:**
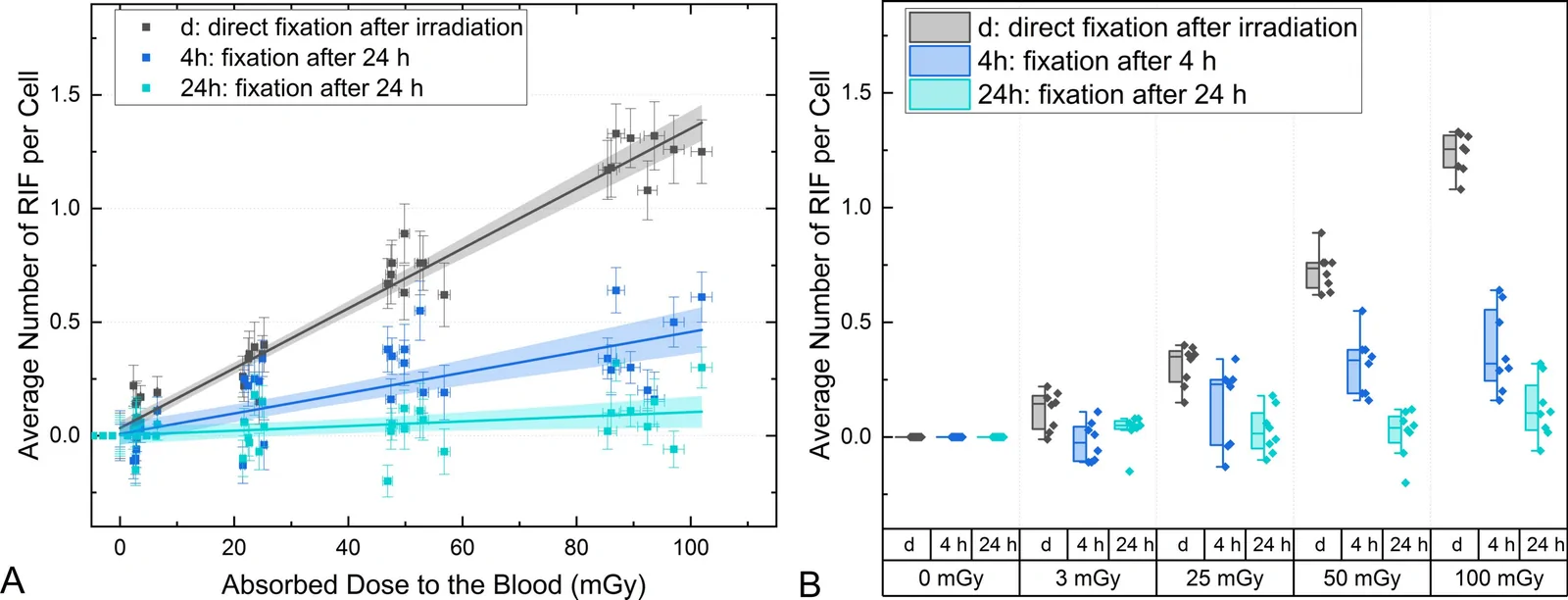
A: Relationship between the absorbed dose to the blood and the average number of RIF per cell, with linear regression lines fitted to pooled data for the respective time points: directly after irradiation (black), 4 h post-irradiation (dark blue), and 24 h post-irradiation (light blue). B: Boxplots showing the average number of RIF per cell for nominal absorbed doses to the blood of 0 mGy, 3 mGy, 25 mGy, 50 mGy, and 100 mGy after internal irradiation with ^177^Lu at three fixation time points: directly, i.e. 0 h after irradiation (d), 4 h post-irradiation (4 h), and 24 h post-irradiation (24 h).

Fig. 2A illustrates the dose–response relationship for RIF at the three different time points (d = directly fixed = 0 h, 4 h and 24 h) after irradiation. As in Part A, a linear fit was applied to the pooled data for each time point. For the directly fixed samples, the slope of (0.0132 ± 0.0005) mGy^-1^ and the coefficient of determination r = 0.98 were similar to the values obtained in Part A. For the later time points, i.e. 4 h and 24 h post-irradiation, the values for the slope and r decreased, suggesting that a linear function is inappropriate for describing the absorbed dose dependence at these time points. All fit parameters are listed in Table 1.

**Table 1:** Parameters of the linear fit of the pooled data of Part A and the linear fits for the three different time points assessed in Part B.

Study Part	Time point	Intercept	Slope (mGy^-1^)	r
A	0 h (directly fixed)	0.0015 ± 0.0154	0.0112 ± 0.0004	0.94
B	0 h (directly fixed)	0.0330 ± 0.0177	0.0132 ± 0.0005	0.98
B	4 h	0.0083 ± 0.0240	0.0045 ± 0.0006	0.77
B	24 h	0.0017 ± 0.0200	0.0010 ± 0.0004	0.38

Table 2 summarizes the absorbed doses from Part B together with the corresponding median numbers of RIF and foci per cell at the three different time points. The average numbers of RIF per cell for the different absorbed doses and time points after irradiation are also represented in the boxplot in Fig. 2B. Directly fixed cell samples irradiated with 3 mGy, 25 mGy, 50 mGy, and 100 mGy displayed significantly higher RIF levels compared to the non-irradiated controls without RIF (Wilcoxon signed-rank test: 3 mGy, p = 0.016; 25 mGy, p = 0.008; 50 mGy, p = 0.008; 100 mGy, p = 0.008). After cell culture for 4 h and 24 h, significantly elevated values relative to control values were still observed at higher doses (Wilcoxon signed-rank test: 100 mGy – 4 h, p = 0.008; 100 mGy – 24 h, p = 0.039; 50 mGy – 4 h, p = 0.008). No elevated average RIF levels were detected in the remaining groups following 4 h or 24 h repair incubation.

**Table 2:** Results of Part B: Absorbed dose to the blood, added activity and average number of RIF and foci per cell. In each case the median value (including the minimum and maximum value) is given.

Nominal absorbed dose to the blood (mGy)	Nominal activity of the ^177^Lu solution added (MBq)	Absorbed dose to the blood (mGy)	Average number of RIF per cell		
			Average number of foci per cell		
			d	4 h	24 h
0	0	0.00	0.00 (0.00-0.00)	0.00 (0.00-0.00)	0.00 (0.00-0.00)
			0.30 (0.14-0.48)	0.32 (0.12-0.60)	0.33 (0.16-0.43)
3	0.30	2.80 (2.36-6.54)	0.15 (-0.01-0.22)	-0.03 (-0.11-0.11)	0.05 (-0.15-0.08)
			0.45 (0.31-0.51)	0.31 (0.18-0.49)	0.38 (0.16-0.48)
25	2.50	23.10 (21.52-25.22)	0.35 (0.15-0.40)	0.23 (-0.13-0.34)	0.02 (-0.10-0.18)
			0.62 (0.36-0.88)	0.48 (0.28-0.61)	0.31 (0.22-0.47)
50	5.00	49.80 (46.89-56.82)	0.74 (0.62-0.89)	0.34 (0.16-0.55)	0.04 (-0.20-0.12)
			1.12 (0.93-1.41)	0.65 (0.47-1.08)	0.40 (0.15-0.78)
100	10.00	90.95 (85.43-101.93)	1.26 (1.08-1.33)	0.32 (0.16-0.64)	0.11 (-0.06-0.32)
			1.48 (1.39-1.80)	0.73 (0.42-0.92)	0.42 (0.29-0.58)

After incubation for 4 h and 24 h, samples irradiated with 50 mGy and 100 mGy showed a significant reduction in average RIF per cell compared to directly fixed samples (t-test) indicating DSB repair. At low (3 mGy, 25 mGy) absorbed doses, however, the differences between directly fixed samples and those after incubation were not significant (Wilcoxon signed-rank test: 3 mGy – d vs. 3 mGy – 24 h, p = 0.109; 25 mGy – d vs. 25 mGy – 4 h, p = 0.078).

Overall, the number of RIF in irradiated samples decreased over time, with 31–44 % of the initial count remaining 4 h after irradiation. After 24 h, the proportion of persistent RIF decreased to 2–10 % of the initial value.

### Results of part B: Modelling of DNA repair

**Fig. 3:**
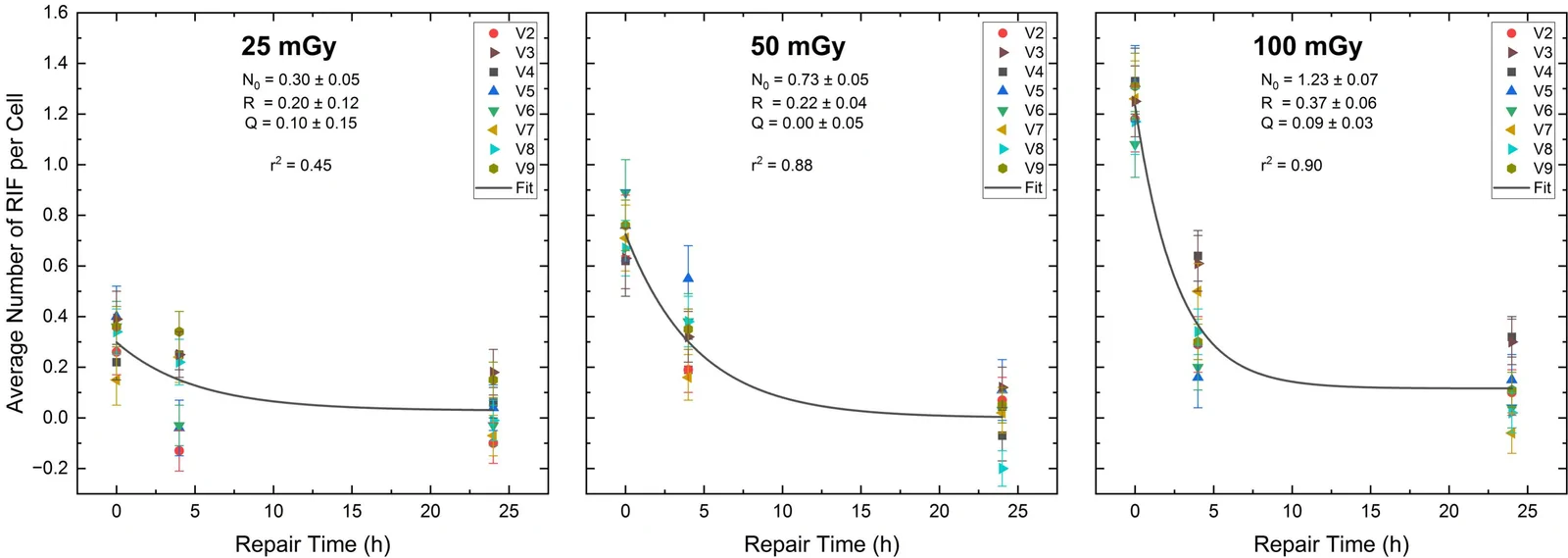
Average number of RIF per cell as a function of repair time after internal irradiation with ^177^Lu for absorbed doses to the blood of 25 mGy, 50 mGy, and 100 mGy. Each data point represents an individual volunteer (V2–V9), and error bars indicate the standard error. The solid line represents a monoexponential repair fit (according to Eq. (2)) to the pooled data, with fitted parameters N_0_ (maximum number of RIF per cell), R (repair rate in h^-1^), and Q (fraction of unrepaired RIF, i.e. the residual damage). Coefficients of determination (r^2^) indicate the goodness of fit.

Fig. 3 shows the time-dependent decrease in RIF for absorbed doses to the blood of 25 mGy, 50 mGy, and 100 mGy. A monoexponential fit according to Eq. (2) was applied to the pooled datasets. Data from the 3 mGy group were excluded, as no reliable fit could be obtained. For the other groups, the fit was successful and the coefficient of determination was r^2^ = 0.45 for 25 mGy, r^2^ = 0.88 for 50 mGy and r^2^ = 0.90 for 100 mGy (Fig. 3). The corresponding fit parameters are summarized in Table 3.

**Table 3:** Percentage of persistent RIF per cell relative to the initial number of RIF per cell in directly fixed samples, along with the monoexponential fit parameters of the pooled datasets: N_0_ (maximum number of RIF per cell), Q (fraction of unrepaired RIF, i.e. residual damage), and R (repair rate in h⁻^1^), including the corresponding r^2^ values. The percentage of persistent RIF per cell was calculated as the mean of the average number of RIF per cell for each time point relative to the mean of the average number of RIF per cell in the directly fixed samples (d) of the corresponding absorbed dose group.

Nominal absorbed dose to the blood (mGy)	Persistent RIF per cell (%)		Fit parameters			
	4 h	24 h	N_0_	R (h^-1^)	Q	r^2^
25	44	9	0.30 ± 0.05	0.20 ± 0.12	0.10 ± 0.15	0.45
50	43	2	0.73 ± 0.05	0.22 ± 0.04	0.00 ± 0.05	0.88
100	31	10	1.23 ± 0.07	0.37 ± 0.06	0.09 ± 0.03	0.90

The RIF repair rate R increased with the absorbed dose to the blood, with values of (0.20 ± 0.12) h⁻^1^ for 25 mGy, (0.22 ± 0.04) h⁻^1^ for 50 mGy, and (0.37 ± 0.06) h⁻^1^ for 100 mGy. The fraction of unrepaired RIF Q did not show a clear trend across the different absorbed doses. The resulting Q values were 0.10 ± 0.15 (25 mGy), 0.00 ± 0.05 (50 mGy), and 0.09 ± 0.03 (100 mGy), which is in agreement with the observation that the proportion of persistent RIF after 24 h was 2-10% of the initial value.

## Discussion

In this study we intended to mimic internal irradiation in patients undergoing RPTs or nuclear medicine diagnostics as closely as possible. We used an *ex vivo* setup that allows well-controlled conditions, such as pre-defined absorbed doses and irradiation durations. A homogeneous irradiation of the blood samples was achieved by incubating them in screwcap tubes on a roller mixer for one hour at 37°C. The activity concentrations added for internal exposure were selected to achieve absorbed doses to the blood in the range of up to nominally 100 mGy after one hour of irradiation, leading to different absorbed dose rates ranging from approx. 3 mGy h^-1^ up to 100 mGy h^-1^. This corresponds to the absorbed doses and absorbed dose rates to the blood in the first few hours after radionuclide administration in most nuclear medicine therapies [9], [12], [16]. In an earlier study including 16 patients, for example, we calculated that the absorbed dose rate to the blood 1 h after administration of 6 GBq [^177^Lu]Lu-PSMA-I&T was 12 mGy h^-1^ in average; 4 h after the administration, the average absorbed dose to the blood was 40 mGy and 109 mGy in total [12]. In this *ex vivo* setup, we kept the irradiation duration fixed at one hour in order to be able to process the largest possible number of samples directly on the day of blood collection. Longer irradiation durations, which might more closely mimic the irradiation *in vivo*, would only have allowed fewer samples to be processed per experimental series, resulting in a decreased accuracy of the calibration curves. Since the absorbed dose rates were consequently not constant, it is also possible that they (and not solely the absorbed dose to the blood) may have influenced the results to some extent.

Using four different radionuclides and calculating the absorbed dose to the blood revealed that the induction of RIF (as a measure of radiation-induced DNA DSBs) exhibits a clear linear absorbed dose dependence in the low dose range < 100 mGy, independent of the tested β- or γ-emitting radionuclide and independent of the volunteer. However, it should be noted that the number of volunteers was limited to only four, which reduces the statistical reliability. Slight radionuclide-dependent differences in DSB induction were calculated by Salas-Ramirez *et al.* using a Monte Carlo simulation model [21]. The calculated DSBs per cell per mGy-values for ^99m^Tc (0.015 ± 0.005), ^90^Y (0.014 ± 0.002), and ^177^Lu (0.011 ± 0.002) fit very well with the slopes of (0.0111 ± 0.0004) mGy^-1^ (Part A) and (0.0132 ± 0.0006) mGy^-1^ (Part B) of the experimental calibration curves determined in this study, showing that theory and experiments correspond precisely within the error intervals. This verifies that the number of radiation-induced γ-H2AX+53BP1 foci is a very suitable measure for the number of radiation-induced DSBs.

For the β^-^-emitters ^131^I and ^177^Lu, an *ex vivo* calibration curve was already established experimentally in 2015 by Eberlein *et al.* using a methodology similar to this study [6]. Two of the four volunteers of Part A in the current study had already participated in the study published in 2015 [6], which implies a certain degree of dependency. Comparing the values of the two slopes, the values determined here are in the same order of magnitude, but slightly lower than the value of (0.0147 ± 0.0006) mGy^-1^ published by Eberlein *et al.* [6]. Possible reasons for this deviation could be an increased statistical power due to the larger subject and radionuclide collective of this study as well as the higher number of samples overall. Furthermore, the uncertainties of the absorbed doses to the blood were taken into account for the curve fitting in this study. Additionally, slight methodological variations, e.g., in the staining procedure may exist.

Compared to the value of the slope of an *in vivo* calibration curve of (0.0117 ± 0.0006) mGy^-1^, which was generated from data collected during the first hours after radioiodine therapies [11], the determined values show a very good agreement. They are also similar to the slopes of *in vivo* calibration curves generated during therapies with [^177^Lu]Lu-DOTATATE/DOTATOC ((0.0127 ± 0.0009) mGy^-1^) [9] and [^177^Lu]Lu-PSMA-I&T ((0.0122 ± 0.0015) mGy^-1^) [12]. This confirms the comparability between the irradiation *ex vivo* as performed here and *in vivo* irradiation in the patient during the first hours after the start of therapy.

In addition to the few studies in which γ-H2AX and/or 53BP1 biomarkers have been used to quantify DNA damage after internal exposure, there is a large number of studies on the dose-dependent induction of DSBs after external irradiation. Calibration curves could be obtained after external *ex vivo* irradiation of blood samples with X- or γ-rays for a similar absorbed dose range as in this study. However, irradiations were performed at higher dose rates of 0.01 Gy min^-1^ to 0.5 Gy min^-1^ and γ-H2AX foci in the lymphocyte samples were evaluated 15 min to 45 min after irradiation. Despite these differences, the comparison with the calibration curves after external irradiation shows a broad agreement: In the range between 20 mGy and 1 Gy, Roch-Lefèvre *et al.* determined a calibration curve with a slope of (0.0107 ± 0.0005) mGy^-1^ after irradiation with ^60^Co-γ-rays with a dose rate of 0.5 Gy min^-1^ [22]. In a study performed by Golfier *et al.* the corresponding value was (0.0074 ± 0.0001) mGy^-1^ after irradiation of blood samples in a CT scanner with a dose rate of 0.3 Gy min^-1^ [23]. Redon *et al.* determined a slope of (0.0115 ± 0.0008) mGy^-1^ for the dose range between 20 mGy and 2 Gy after external irradiation of blood samples [24]. However, these authors observed that in the low dose range, from 20 mGy to 100 mGy, a relatively higher number of γ-H2AX foci is induced, resulting in a slope of 0.0147 mGy^-1^ [24]. Beels *et al.* also observed increased γ-H2AX foci induction in the very low dose range up to 10 mGy (slope of 0.10 mGy^-1^) compared to the dose range from 10 mGy to 500 mGy (slope of 0.011 mGy^-1^), but exclusively after irradiation with X-rays at 0.02 Gy min^-1^ to 0.04 Gy min^-1^ and not in samples irradiated by a ^60^Co-source. After γ-irradiation with dose rates from 0.01 Gy min^-1^ to 0.3 Gy min^-1^, a calibration curve with a slope of 0.0086 mGy^-1^ was obtained [25]. A biphasic behaviour, as described by Redon *et al.* and Beels *et al.* was not observed within the scope of our work. However, since only absorbed doses to the blood in the range of 2.4 mGy to 109.2 mGy were investigated in our current work, it cannot be concluded whether foci induction changes at significantly lower or higher absorbed doses.

Apart from the study by Eberlein *et al.* [6], there is only one other study published in 2019 that describes DNA damage after internal *ex vivo* irradiation of blood samples using different β- and γ-emitting radionuclides, albeit with a different technique: Schmeiser *et al.* used ^177^Lu, ^90^Y, ^18^F and ^68^Ga, each at several different activity concentrations, to quantify DNA damage in blood samples using single cell gel electrophoresis (comet assay) after an extended incubation period of five days for irradiation with ^177^Lu and ^90^Y and 24 h for irradiation with ^18^F and ^68^Ga, respectively. For all radionuclides, an increase in DNA damage was observed with increasing activity concentration in the blood [26]. However, since this study lacks dosimetry and addressed DNA fragment release from nuclei, a direct comparison between the radionuclides and with our and other studies is not possible.

In a recently published study by Mazzitelli-Fuentes *et al.*, the γ-H2AX assay was used to measure absorbed dose and time dependent DNA DSBs in PBMCs after irradiation with radioiodine [17]. In accordance with our results, they found a linear relationship between the number of γ-H2AX foci per cell and the absorbed dose to the blood. In addition, they investigated the influence of irradiation duration and irradiated the blood samples for up to 24 hours, observing an exponential decrease in foci with increasing irradiation duration. Although we analyzed the dependence of the number of foci on the repair time after internal irradiation (and not on the irradiation duration itself), the exponential decrease in foci over time qualitatively matches the observations of Mazzitelli-Fuentes *et al.* well [17].

The DNA repair-associated decline in RIF over time (DSB repair), which is described by an exponential function according to Eq. (2), was also seen after irradiating blood with ^131^I [14] and after mixed α/β-irradiation with ^223^Ra and ^177^Lu [15]. A clear absorbed dose-dependent change in N_0_, describing the initial number of RIF per cell, was observed across all datasets when comparing our results. N_0_ increases with increasing absorbed dose to the blood, which is consistent with the findings after mixed α- and β- irradiation of Strobel *et al.* [15]. At 50 mGy, our N_0_ value (0.73 ± 0.05) was almost identical to that reported by Schumann *et al.* (0.71 ± 0.02) [14]. In contrast, Strobel *et al.* [15] reported higher values at different absorbed doses: (0.81 ± 0.08) at 50 mGy absorbed dose to the blood due to irradiation by β-particles (β-dose) and (0.65 ± 0.06) at 25 mGy β-dose. These discrepancies likely reflect differences in radiation quality and experimental conditions, particularly the presence of an α- and β-emitter mixture during internal irradiation [15]. Despite these variations, all datasets displayed the expected absorbed dose-dependent change in N_0_, with differences in absolute values most likely being due to methodological factors. The repair rate parameter R also showed an increase with higher absorbed doses to the blood across all datasets. The observation could indicate that simple DSBs induced by β-radiation are repaired slower at low absorbed doses. This trend was also seen after irradiation with ^177^Lu, but not after α-particle exposure [15]. This observation aligns with the finding that DSB repair is ineffectively induced at doses in the low mGy range [5] but is stimulated by low ROS levels or higher doses of low LET irradiation [27]. The absence of this effect after α-particle irradiation likely links to the high number of DSBs induced along a single particle track [28], [29]. At 50 mGy, our R-value of (0.22 ± 0.04) h^-1^ is slightly lower than those reported by Schumann *et al.* ((0.28 ± 0.03) h^-1^) [14] and Strobel *et al.* ((0.29 ± 0.13) h^-1^) [15] but remained within the range of uncertainty. In contrast, notable differences were observed for the fraction of unrepaired damage Q. In accordance to Schumann *et al.* (Q = 0.06) [14], our current Q-values remained relatively low at all absorbed doses (0.00–0.10), whereas Strobel *et al.* reported a pronounced increase with decreasing dose (0.14–0.35). This divergence is likely related to the complex DNA damage induced by the high LET contribution in mixed α- and β-irradiation conditions [15]. In general, Eq. (2) appears to be suitable for describing the decline in RIF and thus the repair process of DSBs. However, one limitation is the dependency of the parameters. For example, the amount of residual damage (Q) affects the repair rate (R), so this relationship should be considered when comparing the values. The greater uncertainties in our low-dose measurements further complicate direct quantitative comparisons. Model fitting quality (r^2^) was high at higher doses (up to 0.90) but declined at lower doses.

## Conclusion

Our experiments confirm the applicability of the γ-H2AX+53BP1 DSB focus assay for quantifying the induction and decline of DSBs in PBMCs after internal irradiation in the low-dose range up to approximately 100 mGy. We observed a linear relationship between the absorbed dose to the blood and the number of radiation-induced γ-H2AX+53BP1 foci, which is independent of the radionuclide used. The decline in foci after irradiation follows an exponential function with a faster repair rate with increasing absorbed dose or dose rate. This aligns with previous research suggesting that DNA repair rates are absorbed dose-dependent and reduced at lower absorbed doses.

Overall, this study emphasizes that the administered activity is not the correct measure for estimating and comparing biological responses to exposure to radioactive substances; rather, it is the absorbed dose or dose rate that is important. A better understanding of absorbed dose-response relationships is therefore crucial for optimizing future nuclear medicine therapies.

## Author contributions

U.E., S.Sc., H.S., I.S., and M.L. designed the study and developed the methodology. S.Sc., I.S. and J.H. performed the irradiation of the blood samples and the calculations of the absorbed doses. H.S. performed γ-H2AX+53BP1 staining and foci analysis. All authors analysed and interpreted the data. S.Sc. and I.S. wrote the manuscript. All authors reviewed and revised the manuscript. All authors have read and agreed to the published version of the manuscript.

## Institutional Review Board Statement

The study protocol was presented to the local Ethics Committee of the Medical Faculty of the University of Würzburg, Germany (Az: 165/14). The Ethics Committee approved the study by stating that there were no objections to the conduct of the study. All procedures performed in this study involving human participants were in accordance with the ethical standards of the local Ethics Committee and with the principles of the 1964 Declaration of Helsinki and its later amendments or comparable ethical standards.

## Informed Consent Statement

Informed consent was obtained from all individual participants included in the study.

## CRediT authorship contribution statement

**Sarah Schumann:** Writing – review & editing, Writing – original draft, Visualization, Validation, Supervision, Methodology, Investigation, Formal analysis, Data curation, Conceptualization. **Isabella Strobel:** Writing – review & editing, Writing – original draft, Visualization, Validation, Methodology, Investigation, Formal analysis, Data curation, Conceptualization. **Johanna Hahn:** Writing – review & editing, Methodology, Investigation. **Alberto Meca Zapata:** Writing – review & editing, Methodology, Investigation. **Michael Lassmann:** Writing – review & editing, Validation, Supervision, Project administration, Methodology, Investigation, Funding acquisition, Formal analysis, Conceptualization. **Harry Scherthan:** Writing – review & editing, Validation, Supervision, Project administration, Methodology, Investigation, Funding acquisition, Formal analysis, Data curation, Conceptualization. **Uta Eberlein:** Writing – review & editing, Validation, Supervision, Project administration, Methodology, Investigation, Funding acquisition, Formal analysis, Data curation, Conceptualization.

## Funding

This study was funded by a grant of the Deutsche Forschungsgemeinschaft (DFG; grant nos. LA 2304/3-2, SCHE 350/12-2, EB 639/2-1 and SCHE 350/15-1). The funders had no role in study design, data collection, analysis and interpretation, decision to publish, or preparation of the manuscript. The funders had no role in study design, data collection and analysis, decision to publish or preparation of the manuscript.

## Declaration of competing interest

The authors declare the following financial interests/personal relationships which may be considered as potential competing interests: Uta Eberlein, Harry Scherthan, Michael Lassmann report financial support was provided by Deutsche Forschungsgemeinschaft. Uta Eberlein, Michael Lassmann report a relationship with Novartis that includes: funding grants. Michael Lassmann reports a relationship with Pentixapharm that includes: funding grants. Uta Eberlein: Associate Editor for “Zeitschrift für Medizinische Physik”. Given her role as associate editor, she had no involvement in the peer review of this article and had no access to information regarding its peer review. Full responsibility for the editorial process for this article was delegated to another journal editor. If there are other authors, they declare that they have no known competing financial interests or personal relationships that could have appeared to influence the work reported in this paper.
