# Supplementary material for: DNA damage induction and repair in peripheral blood mononuclear cells induced by internal ex vivo irradiation with β- and γ-emitting radionuclides
Source: Z Med Phys. 2026 Mar 22;36(3):399–406. doi: 10.1016/j.zemedi.2026.03.010 (PMC13589433; doi:10.1016/j.zemedi.2026.03.010)
Supplement: Supplementary file 1 [file mmc1.pdf]

DNA damage induction and repair in peripheral blood mononuclear cells induced by internal *ex vivo* irradiation with β- and γ-emitting radionuclides

Supplementary Material

Suppl. Table 1: S-values S_{Blood} and absorbed dose coefficients d_{Blood} calculated for the geometry of the incubation tubes by using the data published by Hänscheid et al. [1], by Eberlein et al. [2] and by Salas-Ramirez et al. [3].

Radionuclide	S_{Blood} (Gy s^{-1} Bq^{-1} ml)			d_{Blood} (1 h) (mGy ml MBq^{-1})			Reference
	electrons	photons	total	electrons	photons	total	
^{90}Y	1.02E-10	-	1.02E-10	366.80	-	366.80	[1]
^{68}Ga	8.95E-10	2.99E-12	9.25E-11	240.81	8.05	248.86	[1]
^{99m}Tc	2.58E-12	4.18E-13	3.00E-12	8.78	1.42	10.20	[1]
^{177}Lu (Part A)	2.31E-11	1.32E-13	2.32E-11	82.87	0.47	83.34	[2]
^{177}Lu (Part B)	2.22E-11	6.12E-14	2.23E-11	79.74	0.22	79.96	[3]

Suppl. Table 2: Parameters of the individual linear fits for the experimental series of part A and B.

Study part	Radionuclide	Volunteer	Intercept	Slope (mGy^{-1})	r
A	^{177}Lu	V1	0.1660 ± 0.0753	0.0127 ± 0.0018	0.962
A	^{177}Lu	V2	-0.0430 ± 0.0405	0.0089 ± 0.0009	0.990
A	^{177}Lu	V3	-0.0569 ± 0.0418	0.0125 ± 0.0012	0.997
A	^{177}Lu	V4	0.0229 ± 0.0551	0.0123 ± 0.0013	0.977
A	^{90}Y	V1	-0.0141 ± 0.0644	0.0122 ± 0.0019	0.969
A	^{90}Y	V2	0.0173 ± 0.0434	0.0110 ± 0.0016	0.997
A	^{90}Y	V3	0.0591 ± 0.0433	0.0098 ± 0.0016	0.974
A	^{90}Y	V4	0.0295 ± 0.0799	0.0180 ± 0.0028	0.969
A	^{68}Ga	V1	-0.0954 ± 0.0511	0.0122 ± 0.0014	0.973
A	^{68}Ga	V2	-0.0259 ± 0.0429	0.0099 ± 0.0012	0.999
A	^{68}Ga	V3	-0.0225 ± 0.0400	0.0106 ± 0.0010	0.995
A	^{68}Ga	V4	-0.0554 ± 0.0647	0.0112 ± 0.0018	0.974
A	^{99m}Tc	V1	0.1082 ± 0.0510	0.0102 ± 0.0012	0.971
A	^{99m}Tc	V2	0.0173 ± 0.0437	0.0111 ± 0.0012	0.994
A	^{99m}Tc	V3	0.0563 ± 0.0402	0.0118 ± 0.0010	0.988
A	^{99m}Tc	V4	0.0986 ± 0.0530	0.0102 ± 0.0013	0.967
B	^{177}Lu	V2	0.0527 ± 0.0560	0.0129 ± 0.0016	0.985
B	^{177}Lu	V3	0.0605 ± 0.0560	0.0118 ± 0.0014	0.995
B	^{177}Lu	V4	0.0378 ± 0.0579	0.0131 ± 0.0023	0.971
B	^{177}Lu	V5	0.0012 ± 0.0665	0.0143 ± 0.0017	0.999
B	^{177}Lu	V6	0.0707 ± 0.0673	0.0123 ± 0.0018	0.964
B	^{177}Lu	V7	-0.0546 ± 0.0642	0.0136 ± 0.0017	0.987
B	^{177}Lu	V8	0.0114 ± 0.0500	0.0138 ± 0.0015	0.999
B	^{177}Lu	V9	0.0346 ± 0.0451	0.0145 ± 0.0014	0.996

References

[1] Hänscheid H, Fernandez M, Eberlein U, Lassmann M. Self-irradiation of the blood from selected nuclides in nuclear medicine. Phys Med Biol 2014; 59: 1515–31.

[2] Eberlein U, Peper M, Fernandez M, Lassmann M, Scherthan H. Calibration of the gamma-H2AX DNA double strand break focus assay for internal radiation exposure of blood lymphocytes. PLoS One 2015; 10: e0123174.

[3] Salas-Ramirez M, Lassmann M, Eberlein U. GATE/Geant4-based dosimetry for ex vivo in solution irradiation of blood with radionuclides. Z Med Phys 2023; 33: 46–53.
